# The role of ECG screening in primary care; a call for collaboration between general practitioner and cardiologist

**DOI:** 10.1007/s12471-020-01410-4

**Published:** 2020-03-10

**Authors:** G. A. Somsen

**Affiliations:** Cardiologie Centra Nederland, Amsterdam, The Netherlands

General practitioners in the Netherlands are motivated by guidelines and scientific publications to perform electrocardiography in their practices, mainly to detect atrial fibrillation, especially in high-risk patients. Since electrocardiography screening is increasingly being used in various programmes, such as cardiovascular prevention and diabetes coaching, information is needed with respect to the diagnostic yield of a broader use of ECG screening. Interestingly, the article by Van den Nieuwenhof et al., published in the current issue of the Netherlands Heart Journal, provides valuable information on the added value of the electrocardiogram (ECG) at the general practitioner’s office [1].

In this study, ECGs performed for a specific indication (i.e. complaint driven) show a higher diagnostic yield than routinely performed ECGs. The authors report an overall modest yield of ECG use in the general practitioner’s office. However, GPs detected abnormalities, such as atrial fibrillation, in 13% (*n* = 111) of the routinely performed ECGs. This could have major therapeutic consequences as starting anticoagulation can prevent stroke in patients with atrial fibrillation. In addition, previous myocardial infarction and intraventricular conduction disease was detected in 87 of the 111 cases. This may have diagnostic as well as therapeutic consequences for these patients since these ECG findings warrant secondary prevention and are associated with cardiomyopathy, respectively.

The accuracy of ECG reviewing was moderate. Since only a relatively small number of general practitioners performs ECGs and only 14 ECGs are performed per month per general practitioner, it can be anticipated that increasing the accuracy is not easily achieved.

As holds for many studies, the current study also gives rise to several questions.

First, how can the accuracy of ECG reviewing by the general practitioners be improved? Since the prevalence of cardiovascular disease will grow, an increasing number of patients may benefit from ECG screening, which means that more general practitioners need to be educated. ECG reading skills require repeated practice and feedback. It takes more than 3 hours and over 70 cases of ECG training to obtain a diagnostic accuracy of 85% for detecting arrythmia and conduction disorders using a single-lead ECG [2]. In addition, the relatively low frequency of ECG use per general practitioner, compared with emergency physicians and cardiologists, raises the question of whether acquired knowledge on ECG reviewing will remain accurate over time. Structured and repeated training combined with automated reviewing of ECGs based on machine learning, with higher accuracy than the current algorithms, may solve this problem.

Second, do the detected ECG abnormalities result in the correct diagnostic procedures and are these outcomes influencing clinical decision making, which may potentially improve prognosis in these patients? A patient with an ECG abnormality, which may indicate cardiac pathology, often needs additional diagnostics, such as echocardiography, MRI or coronary CT angiography. These are only available in cardiology clinics. To enable effective referral to a cardiologist, the general practitioner should be trained and equipped with protocols. These protocols need to be developed and evaluated in an interdisciplinary collaboration between general practitioners and cardiologists.

Third, as holds for all screening programmes, what will be the effect of false positive and false negative outcomes? The first may lead to unnecessary testing and undue anxiety in patients while the latter will erroneously comfort patients and doctors. For example, a single measurement in time may leave a considerable number of patients with paroxysmal atrial fibrillation undetected. Prolonged (continuous) ECG registration using mobile ECG devices identifies 8 times more atrial fibrillation in post-stroke patients compared with short ECG monitoring [3]. Interestingly, the use of these devices by consumers leads to a high number of false positive results, as has recently been shown by the Apple Heart Study [4].

Fourth, is ECG screening at the general practitioner’s office cost-effective? Results of a systematic review seem to suggest that ECG screening for atrial fibrillation in primary care is cost-effective [5]. However, evaluation of the long-term outcome of ECG screening and interpretation of 12-lead ECGs by the general practitioner is still needed. In addition, we also need to analyse the cost-effectiveness of screening with 12-lead ECGs, which enables detection of other cardiac pathology (i.e. conduction disorder, ventricular hypertrophy, previous myocardial infarction, etc.), and the health-related yield of ECG screening in specific populations at the general practitioner’s office (i.e. high cardiovascular risk and diabetes).

To increase diagnostic accuracy and improve adequate patient referral using ECG screening in the general practitioner’s office several measures can be taken. First and foremost, lowering the threshold for interaction between general practitioner and cardiologist. This is increasingly facilitated by digital platforms and local partnerships. The general practitioner sends ECGs that are difficult to interpret to the cardiologist, who reviews the ECGs and provides diagnostic and therapeutic advice. Furthermore, these local and/or national partnerships need to address other topics such as education, screening indication and referral guidelines.

This study strengthens the need for better collaboration between general practitioners and cardiologists to improve cardiovascular care. Since early detection of cardiac pathology may lead to prompt treatment, the use of ECG screening in the general practitioner’s office may potentially reduce morbidity and mortality. However, additional randomised trials are needed to demonstrate the cost-effectiveness as well as the morbidity and mortality effects of early detection and treatment of a variety of cardiac diseases in various patient populations using ECG screening in primary care.
